# COVID-19 infection segmentation using hybrid deep learning and image processing techniques

**DOI:** 10.1038/s41598-023-49337-1

**Published:** 2023-12-20

**Authors:** Samar Antar, Hussein Karam Hussein Abd El-Sattar, Mohammad H. Abdel-Rahman, Fayed F. M. Ghaleb

**Affiliations:** Computer Science Division, Department of Mathematics, Faculty of Science, Ain Shams University, Abbassia, Cairo, 11566 Egypt

**Keywords:** Diseases, Health care, Medical research

## Abstract

The coronavirus disease 2019 (COVID-19) epidemic has become a worldwide problem that continues to affect people’s lives daily, and the early diagnosis of COVID-19 has a critical importance on the treatment of infected patients for medical and healthcare organizations. To detect COVID-19 infections, medical imaging techniques, including computed tomography (CT) scan images and X-ray images, are considered some of the helpful medical tests that healthcare providers carry out. However, in addition to the difficulty of segmenting contaminated areas from CT scan images, these approaches also offer limited accuracy for identifying the virus. Accordingly, this paper addresses the effectiveness of using deep learning (DL) and image processing techniques, which serve to expand the dataset without the need for any augmentation strategies, and it also presents a novel approach for detecting COVID-19 virus infections in lung images, particularly the infection prediction issue. In our proposed method, to reveal the infection, the input images are first preprocessed using a threshold then resized to 128 × 128. After that, a density heat map tool is used for coloring the resized lung images. The three channels (red, green, and blue) are then separated from the colored image and are further preprocessed through image inverse and histogram equalization, and are subsequently fed, in independent directions, into three separate U-Nets with the same architecture for segmentation. Finally, the segmentation results are combined and run through a convolution layer one by one to get the detection. Several evaluation metrics using the CT scan dataset were used to measure the performance of the proposed approach in comparison with other state-of-the-art techniques in terms of accuracy, sensitivity, precision, and the dice coefficient. The experimental results of the proposed approach reached 99.71%, 0.83, 0.87, and 0.85, respectively. These results show that coloring the CT scan images dataset and then dividing each image into its RGB image channels can enhance the COVID-19 detection, and it also increases the U-Net power in the segmentation when merging the channel segmentation results. In comparison to other existing segmentation techniques employing bigger 512 × 512 images, this study is one of the few that can rapidly and correctly detect the COVID-19 virus with high accuracy on smaller 128 × 128 images using the metrics of accuracy, sensitivity, precision, and dice coefficient.

## Introduction

In March 2020, the World Health Organization (WHO) announced the spread of the coronavirus disease 2019 (COVID-19) and characterized it as a pandemic caused by the severe acute respiratory syndrome coronavirus (SARS-CoV-2). Millions of individuals worldwide are at risk for SARS-CoV-2 infection, which can be fatal if they are left untreated. The WHO recorded 131,309,792 cases globally as of April 5, 2021, with 2,854,276 fatalities and a mortality rate of more than 2%^1^. COVID-19 is transmitted through respiratory droplets found in an individual’s coughs or sneezes, and these droplets might transmit the infection further by contaminating the nearby surfaces. Individuals infected with COVID-19 may experience mild to severe respiratory illnesses and may require assistance with breathing^2,3^. As COVID-19 is a rapidly spreading pandemic, early detection is essential for disease control, effective management, and halting the spread of infection. Diverse methods, including serological, nucleic acid, antigen, and ancillary tests^4^, where each of them has particular uses in hospitals and healthcare, may be used to detect COVID-19. Molecular techniques such as reverse transcription polymerase chain reaction (RT-PCR) are the most frequently used tests. Owing to its accurate detection rate, high sensitivity, and specificity, RT-PCR is frequently recognized as the gold standard for COVID-19 detection^5^. RT-PCR is the most common approach for confirming infected cases because it can identify SARS-CoV-2 from respiratory specimens obtained by nasopharyngeal or pharyngeal swabs within 4–6 h. However, a major problem in many nations around the world is the lack of RT-PCR test kits and also the specialized equipment required for testing. In addition, there is a shortage of trained radiologists in some areas, making it difficult to scale up this approach. Furthermore, lot studies (for instance^6,7^) have shown that RT-PCR assays have substantial false-positive and false-negative rates. This is thought to be mostly due to the fast changes and genetic diversity of the coronavirus^8^. This is thought to be mostly due to the fast changes and genetic diversity of the coronavirus^7^. COVID-19 appears to be a lung infection; therefore, one of the best ways to automatically identify COVID-19 is by using medical imaging techniques such as computed tomography (CT) scans and chest X-ray (CXR) images. Since COVID-19 presents as a lung infection, medical imaging methods, including computed tomography (CT) scans and chest X-ray (CXR) images^9,10^ are among the best approaches to automatically diagnosing COVID-19. Because so many laboratories and institutes have access to these techniques, researchers used CXR images and CT scan images to identify COVID-19. However, there are numerous difficulties in segmenting infected regions from CT slices. Moreover, these techniques are less accurate than RT-PCR and can result in false positives or missed early-stage infections. These challenges highlight the need for new and innovative approaches to improve the COVID-19 diagnosis. Deep Learning (DL) techniques have shown promising potential in this area due to their ability to analyze enormous amounts of data rapidly and reliably^11–17^. Even prior to the COVID-19 epidemic, DL techniques demonstrated noteworthy performance in a variety of research applications, including object recognition^18,19^, face recognition^20^, object tracking^21^, steganography^22^, and medical image analysis^23,24^, as well as in disease detection, such as skin cancer detection, breast cancer detection, lung segmentation and detection of malaria on blood smear images^25–29^. In medical imaging analysis, issues with COVID-19 detection can be interpreted as classification and segmentation to identify and detect abnormal features and regions of interest (ROIs) using DL techniques. The scholars in^7^ detected COVID-19 using a DL algorithm and achieved a high performance, while the authors in^30^ used a DL method to extract features from COVID-19 X-ray images and then classified the features using a support vector machine (SVM). On the other hand, the authors of^31^ proposed an explainable deep learning approach and used 2482 CT scan images of 120 people to classify COVID-19-infected patients and healthy people, and the accuracy of this study was 97.38%. Furthermore, the authors of^12^ developed a diagnostic system for COVID-19 detection, where they used morphological dilation and a histogram thresholding approach to segment the lung CT scan images. Also, that study utilized a convolution layer with various filter widths to build the CNN architecture, and a softmax layer was employed to classify COVID-19 images. The accuracy of that CNN model was 88.3%, while the ground-class opacity in COVID-infected areas was not segmented despite segmenting the lung CT scan images. Lastly, a generalized approach for segmenting and categorizing lung CT scan images was addressed in^13^, and they segmented lung CT scan images using the U-Net approach and achieved a higher performance accuracy of 98.98%; however, the ground-class opacities caused by inflammation were not segmented properly. In the body of this current research, multiple studies that concentrate only on the detection of COVID-19 using DL techniques are outlined, while only a few studies that concentrate on the segmentation of the COVID-19 region and classification using image processing and DL techniques are showcased. This paper consequently addresses these issues using U-Net to detect and quantify COVID-19 infection from clinical CT scan images taken from a freely available dataset of chest CT scan images^32^. The structure of this paper is as follows: "Literature review" section offers a more comprehensive review of the literature. "Contribution" section highlights the benefits of our contribution over existing work. "Basic notions" section presents the theoretical background. The "Prior research" section examines some of the earlier studies on COVID-19 detection. Our proposed technique and datasets are provided in "Datasets and Methodology" sections. The "Result analysis and discussion" section of the paper includes results, comparative analysis, an ablation study, and limitations. "Conclusion" section concludes this paper.

## Literature review

Despite some claims to the contrary, COVID-19 is resurfacing, so early COVID-19 illness identification is necessary for controlling this outbreak. For this reason, medical imaging processing techniques have recently become widely used for the automated identification of COVID-19, as CT scan and X-ray images are analyzed using artificial intelligence technologies^33^. In this regard, several methods have been proposed recently for the detection and segmentation of COVID-19 infection in the lungs using chest X-rays and CT scans. In order to identify COVID-19 using these methods, feature extraction is a crucial step either using manual techniques or deep learning techniques^12–17,23,34,35^. For instance, the authors of^14^ employed manual feature extraction methods to calculate frequency and spatial features from X-ray images to build a feature vector of 256 elements. After that, they used Principal Component Analysis (PCA) to select the most significant features, which were then used to train and test a multilayer perceptron (MLP) network to classify healthy, pneumonia, and COVID-19 cases. Thus, scholars have been motivated to carry out more research in deep learning due to the absence of manual feature extraction and the existence of an end-to-end architecture. Accordingly, hybrid approaches have been used to detect COVID-19. For instance, the scholar's in^36^ discuss various deep learning techniques used for COVID-19 detection and diagnosis, such as convolutional neural networks (CNNs), recurrent neural networks (RNNs), and hybrid models. These approaches use the pre-trained convolution neural network (CNN) models as feature extractors and the classical machine learning algorithms in the classification process as in^11,37–40^. For example, the COVID-19 CT-scan binary classification work was handled by the authors of^40^ using four potent pre-trained CNN models: VGG16, DenseNet121, ResNet50, and ResNet152. This approach uses a FastAI ResNet framework to automatically choose the optimum architecture from CT scans. To get around the lengthy training period, transfer learning strategies were also employed, since transfer learning performs best when there are few available training data sets. Another method proposed in^11^ comparing the effectiveness of three pre-trained CNN models, AlexNet, ResNet50, and SqueezeNet, with that of three machine learning classifiers, Nave Bayes, Bagging, and Reptree, to categorize the chest CT images into two image classes, namely COVID and non-COVID. The three CNN models were trained using the transfer learning strategy as well. According to their research, the Naive Bayes classifier had the best accuracy (97%), while ResNet50, one of the three CNN models, had the highest accuracy (99.1%). As a result, they reported that while classifying COVID-19 chest CT scan images, deep learning networks outperformed machine learning approaches. On the other hand, because of their high sensitivity, specificity and correct prediction rate, molecular methods like reverse transcription-polymerase chain reaction (RT-PCR) are frequently recognized as the gold standard for identifying COVID-19^5^. The speed and effectiveness of screening suspected cases, however, are hindered by a lack of resources for performing RT-PCR assays. Furthermore, several studies have shown that RT-PCR tests have high false positive and false negative rates^6,7^. The fast mutations and genetic variety of COVID-19 are thought to be the main cause of this. Several studies^41–43^ have identified COVID-19 by identifying features in its genomic sequence in addition to using molecular methods and medical imaging modalities. These methods use a variety of genomic signal mapping algorithms to convert genome sequences into genomic signals. To create useful systems that can detect COVID-19, these signals were analyzed using digital signal processing technologies. In another study, COVID-19 was identified using genomic image-processing (GIP) methods^44^. GIP is an area of bioinformatics that connects bioinformatics with image processing techniques.

## Contribution

Since COVID-19 is a novel pandemic, only a limited number of datasets are publicly available. The size of the data is a key factor in the performance of any DL model. Data augmentation is a useful approach to overcoming the dataset's size limitation and preventing over-fitting or under-fitting issues. The majority of the methods employed traditional augmentation techniques, such as rotation, shearing, scaling, and horizontal and vertical translations, among others, or a generative adversarial network (GAN)^45^ to overcome the issue of reliance on original data, which may occurs using traditional augmentation techniques. Although the majority of most methods have sought to employ different data augmentation techniques to make up for the lack of a dataset, the efficacy of data augmentation in real-life and live images for the detection of COVID-19 remains unclear. Furthermore, general data expansion methods provide visuals that strongly resemble the original images. In our method, we didn't use any augmentation methods and instead employed various pre-processing image processing techniques to split the image into red, green, and blue (RGB) channels, which are fed into three separate U-Net for each RGB image channel. Consequently, the original dataset—3138 CT images—is immediately enlarged into 3138 × 3 without the need for any augmentation methods. Furthermore, to provide a difference in the information passed to the U-Net architecture, which gives more information about the infection, this division contributes to the infection detection by using a large amount of information instead of the grey image, which contains only one channel. The proposed system merges the prediction results of the three RGB image channels to present its final result. The basic idea of the proposed algorithm is to map scalar values of the given CT images to colors (e.g., RGB image channels in our case) and use the heat-map as a data visualization tool. These colors can be used to highlight features in the given images, such as bone density, tissue density, or blood flow given in^27^. In the case of COVID-19, infected individuals often exhibit a fever, which can be detected using thermal imaging and represented as a heat-map^46–48^. Heat-maps are graphical representations used to identify the region of interest in data by observing how colors changed. In the current study, heat-map is applied on the lungs images to visualize the lung part in RGB color space to give more details that facilitate the detection of infection inside the lung. Every channel is passed into U-Net architecture then before the classification the segmented result is merged and passed into 1 × 1 convolution layer for the classification. The three RGB channels were employed to expand the feature information, which enhanced infection prediction. The proposed technique can detect COVID-19 with a 99.71% accuracy rate and improved sensitivity and specificity. The proposed approach possesses the following merits:

1. It avoids the use of data augmentation to overcome the limited size of the dataset, which is commonly applied by most of the previous methods.
2. It uses a small image size to train the model to increase the speed of the training and testing instead of using large images, as commonly used.
3. It uses RGB image channels to predict COVID-19 infection.
4. It merges the results of the COVID-19 prediction of three U-Nets based on the image channel, which increases the prediction accuracy.
5. The different number of filters in the convolution block in the proposed approach reduces the total number of parameters, produces accurate results, and increases the running speed of the model.

## Basic notions

### Convolution neural networks (CNNs)

**Figure 1:**
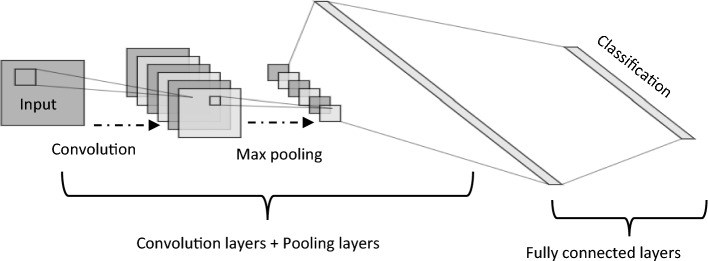
CNNs structure.

One of the most important networks in the field of DL is the convolutional neural networks (CNNs), which are used for a variety of classification problems^49,50^. CNN's success is largely due to its in-built capacity to automatically extract features from input data without operator intervention^51^. One of the major advantages of using CNNs over other neural networks is that they can deal with 2D image data, so we do not need to flatten the input images to 1D, which helps in retaining the “spatial” features of the images. Hierarchical feature representation can be learned automatically from data, which is a multilevel representation from pixels to high-level semantic features learned using a hierarchical multi-stage structure. CNN may be thought of as a series of convolution layers interspersed with nonlinearity and pooling layers that translate activations received at one end into activations received at the other end using a loss or error function. The loss function is used to calculate the error between a single prediction and its associated actual value, so we become able to evaluate the effectiveness of a specific classification function in classifying certain data points in our dataset as "good" or "poor". A general representation of CNNs architecture for image classification is generally composed of four layers: input, convolution, pooling (sub-sampling) and fully connected as demonstrated in Fig. 1.

**Figure 2:**
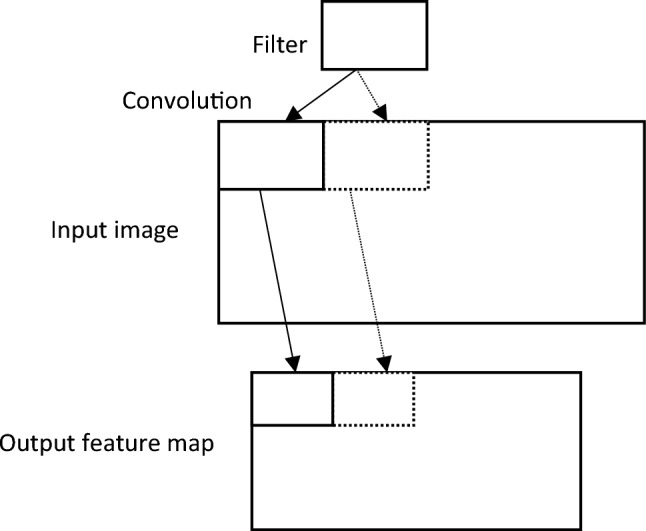
An illustration of a convolution between an image's input and a filter.

**Figure 3:**
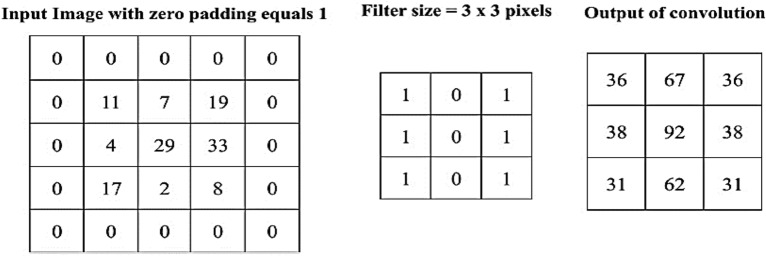
An example of a filter applied to a two-dimensional input image to produce a feature map.

The input layer stores the pixel values of the input images. The image is divided into receptive fields that are fed into a convolutional layer. Receptive fields are areas of the visual field where a single neuron is activated in response to a stimulus. The features of the input image are extracted using a convolution layer. The convolution layers are based on the term "convolution,” which is a mathematical operation performed on two variables (f * g) to produce a third variable. These layers are responsible for identifying low-and high-level complex features in each input. A group of parameters, called hyperparameters, is associated with a convolution layer: filter size, stride, and zero-padding^51^. The hyperparameters are constants whose values must be known before the models can be built. For instance, stride is the number of units in which the filter slides over an input image. A convolutional layer is applied by taking an image as an input matrix of pixels and then applying learnable filters (or kernels) of a fixed size, that is, to each block of the input matrix (see Fig. 2 as an example). A kernel convolves images using a specific set of weights by multiplying its elements with the corresponding elements of the receptive field. Receptive fields are the area of the visual field where a single neuron is activated in response to a stimulus. These multiplications are summed, and the process is repeated for every location in the input volume. Figure 3 depicts what occurs when a filter is applied to an input image with a stride. Convolution produces “feature maps,” which are collections of several features. Consequently, a pooling layer (e.g., max pooling) is used to reduce the dimensionality of each feature map, but it holds the most critical data. The system linearly performs computations until the convolutional layer is reached. The selection of an appropriate activation function, such as Sigmoid, the Rectified Linear Unit (ReLU), and variants of ReLU, is used to introduce nonlinearity in the system. For instance, the purpose of ReLU is to replace negative activation with a 0. The architecture of each CNN had a fully connected layer at the end. Inside this layer, each neuron is connected to all neurons of the previous layer, which is the so-called FC approach. It was used as the CNN classifier.

**Figure 4:**
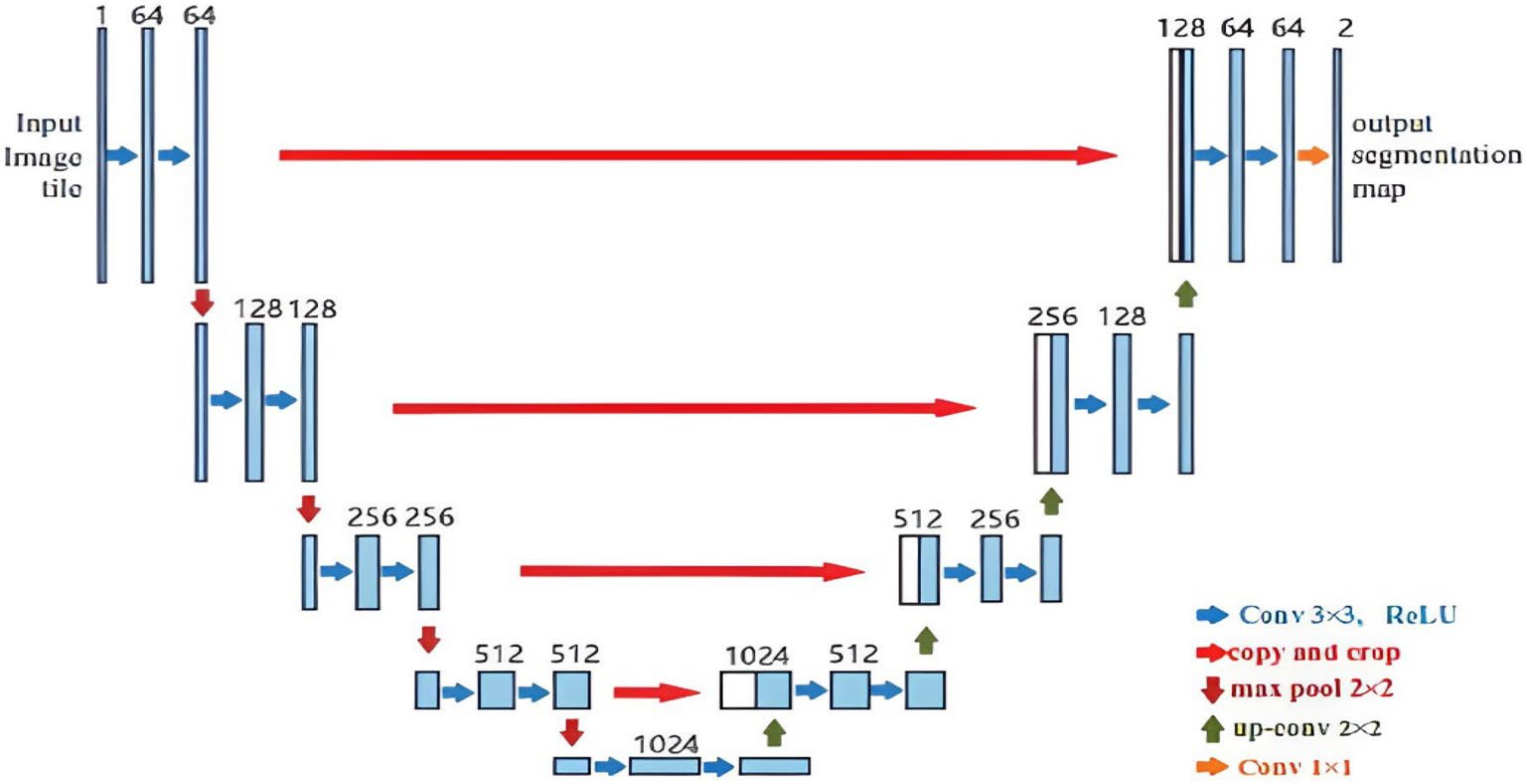
U-Net architecture^53^.

- *U-Nets* U-Net is a convolutional neural network architecture developed for biomedical image segmentation tasks^52^. It was originally proposed by Ronneberger et al.^53^ for biomedical image processing. The name "U-Net" comes from the shape of the network, which resembles the letter "U.". The U-Net architecture is based on the CNN encoder and decoder approaches. The encoder is responsible for the encoding context by using CNN’s typical architecture of alternating convolution and pooling operations. It is composed of five blocks, each of which is composed of two convolutional layers, and uses a ReLU activation function to provide network nonlinearity. These blocks produce feature maps through a convolution process. One max-pooling layer then reduces the size of these feature maps while simultaneously increasing the number of layers per block, allowing the architecture to effectively learn complex structures. By employing transposed convolution (deconvolution) procedures to create the segmentation mask of the picture, the decoder component is responsible for decoding the information, enabling exact localization. It also consists of five blocks, each of which is made up of two convolutional layers, which also use the ReLU as the activation function; one up-sampling layer, which is in charge of reverting the max-pooling operation to restore the feature maps to their original size in the network; and a skip connection, which combines the up-sampled features with high-resolution encoded features from the encoder part. According to Zhou et al.^54^, skip connections can aid gradient propagation in deep networks by reducing the likelihood of gradient dispersion, which enhances segmentation performance. Figure 4 illustrates the overall architecture of the U-Net model.

### Prior research

In recent years, several illnesses have been monitored using medical image-processing techniques^55^. The development of DL and artificial intelligence technologies, which have become popular methods for the identification and segmentation of various medical issues, has accelerated this field's advancement^23,33,52^. In the last few months, several approaches have been proposed for the detection and segmentation of the lungs’ COVID-19 infection using chest X-rays and CT scans. The proposed approaches can be divided into three groups: (1) techniques for classification, (2) techniques for segmenting diseased regions, and (3) diagnostic systems that can handle both tasks. The second group comprised the main topic of this study. For instance, under classification techniques, the authors of^56^ developed a CNN model-based binary classifier to detect COVID-19 from chest CT scan images. Wang et al.^57^ developed COVID-Net, a densely connected deep convolutional neural network architecture that scored 93.3% in a test of accuracy for the purpose of detecting COVID-19 instances from chest X-ray images. Besides, Ahuja et al.^58^, presented a three-phase detection model using deep transfer learning to increase detection accuracy. On the other hand, a hybrid model using transfer learning has also been discussed in^39^ using CT scans to detect COVID-19, and other researchers used infection segmentation techniques^59–65^, the author of^59^, for instance, developed a novel deep network named "Inf-Net" to automatically identify sick areas from chest CT slices. That approach is built around a parallel partial decoder that combines high-level characteristics to produce a world map. They employed a modest dataset for their investigation that had 100 CT-labeled images and a die score of 0.682. Another study was conducted by^60^ who suggested a unique method for detecting COVID-19 characteristics in chest X-ray images by combining CNN and VGG19. They employed a dataset of eighty-seven chest X-ray images linked to twenty-five cases in their investigation and found that it had 96.93% accuracy, 57.14% sensitivity, and 99.2% specificity. Using CT imaging, the authors of^61^ created a diagnostic system based on deep learning methods to identify and quantify COVID-19 infection and pneumonia screening. In addition, the authors of^62^ developed three standards for lung and infection segmentation based on 70 annotated COVID-19 cases. Although numerous authors have split lung CT images, ground-class opacity in COVID-19-infected regions caused by inflammation has not been segmented effectively in the current literature. A small number of studies have focused on the segmentation and classification of the COVID-19 area using image processing and DL techniques, whereas the majority of recent research has only focused on the detection of COVID-19 using DL approaches. The current study tackles these problems by pre-processing the input images with thresholds to exclude infections and coloring the images with a heat-map tool, also it uses U-Net to identify and quantify COVID-19 infections using clinical CT scan images.

## Datasets and methodology

This section presents in-depth information on our proposed methodology and the datasets that are used to train and evaluate our proposed approach.

### Datasets

**Figure 5:**
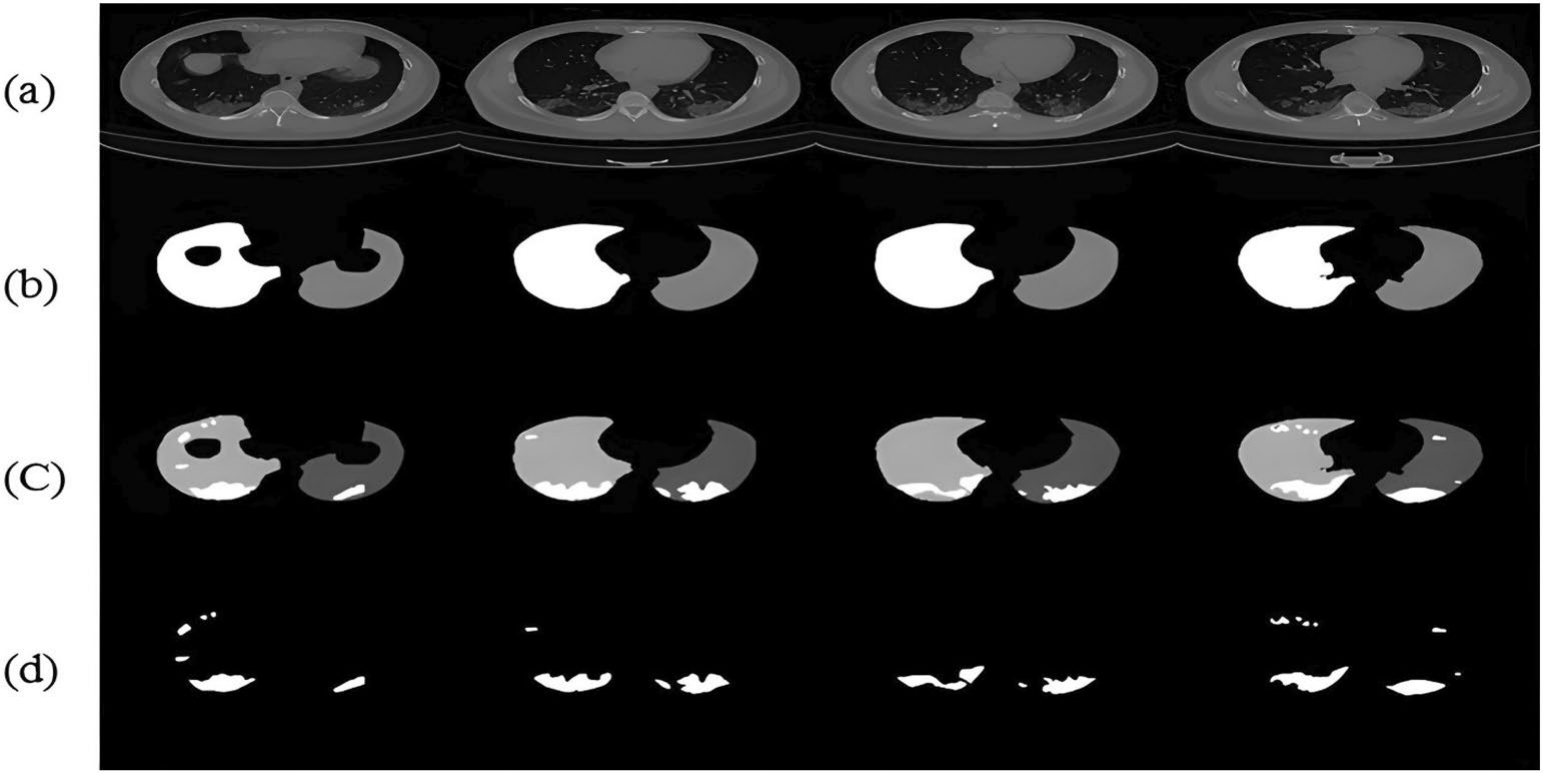
Four sample images from the used dataset. First row (**a**): original CT lung images. Second row (**b**): sample of lung masks. Third row (**c**): sample of infection and lung mask together. Fourth row (**d**): infection masks.

The size of the data has a significant impact on how well any deep learning model performs. Because COVID-19 is a new pandemic, only a few datasets with a small number of samples are available to the public. There are still not enough labeled CT scan images, despite the rise in patients with COVID-19 infection and related volumetric CT scan images. To the best of our knowledge, the COVID-19 CT scan public Kaggle dataset repository (https://www.kaggle.com/datasets/andrewmvd/covid19-ct-scans)^32^ is the first publicly available data-efficient learning benchmark for medical image segmentation, hence we used it in this study to implement our proposed algorithm and train the network. This dataset includes twenty CT images of individuals who were diagnosed with COVID-19, together with expert-made lung segmentations and infection diagnoses. Lung infections vary in percentage, from 0.01 to 59%. It consists of approximately 3138 lung CT images that have been labeled, segmented, and verified by skilled radiologists, along with their correspondent lung CT scan images, their corresponding lung masks, infection masks, and a superposition of the two masks, in addition to 20 axial CTS of patients with COVID-19. Figure 5 displays samples of the employed datasets. Two radiologists classify the left lung, right lung, and infections; a more experienced radiologist confirms the labels. The left lung, right lung, and infection were firstly delineated by junior annotators with 1–5 years' experience and then refined by two radiologists with 5–10 years' experience, and finally all the annotations were verified and refined by a senior radiologist with more than 10 years' experience in chest radiology. In the work of^32^, the dataset was manually annotated and obtained from the Corona-Case Initiative and Radiopaedia (https://radiopaedia.org/ and coronacases.org, respectively). All the annotations are publicly available at https://zenodo.org/record/3757476 with a CC-BY-NC-SA license.

### Methodology

Our proposed approach is presented in detail in this section. The methodology of our proposed approach includes the following stages: The first stage is to acquire patient data in CTS imaging format; the second is data pre-processing; and the third is training and classification using the pre-processed data.

### Data pre-processing

**Figure 6:**
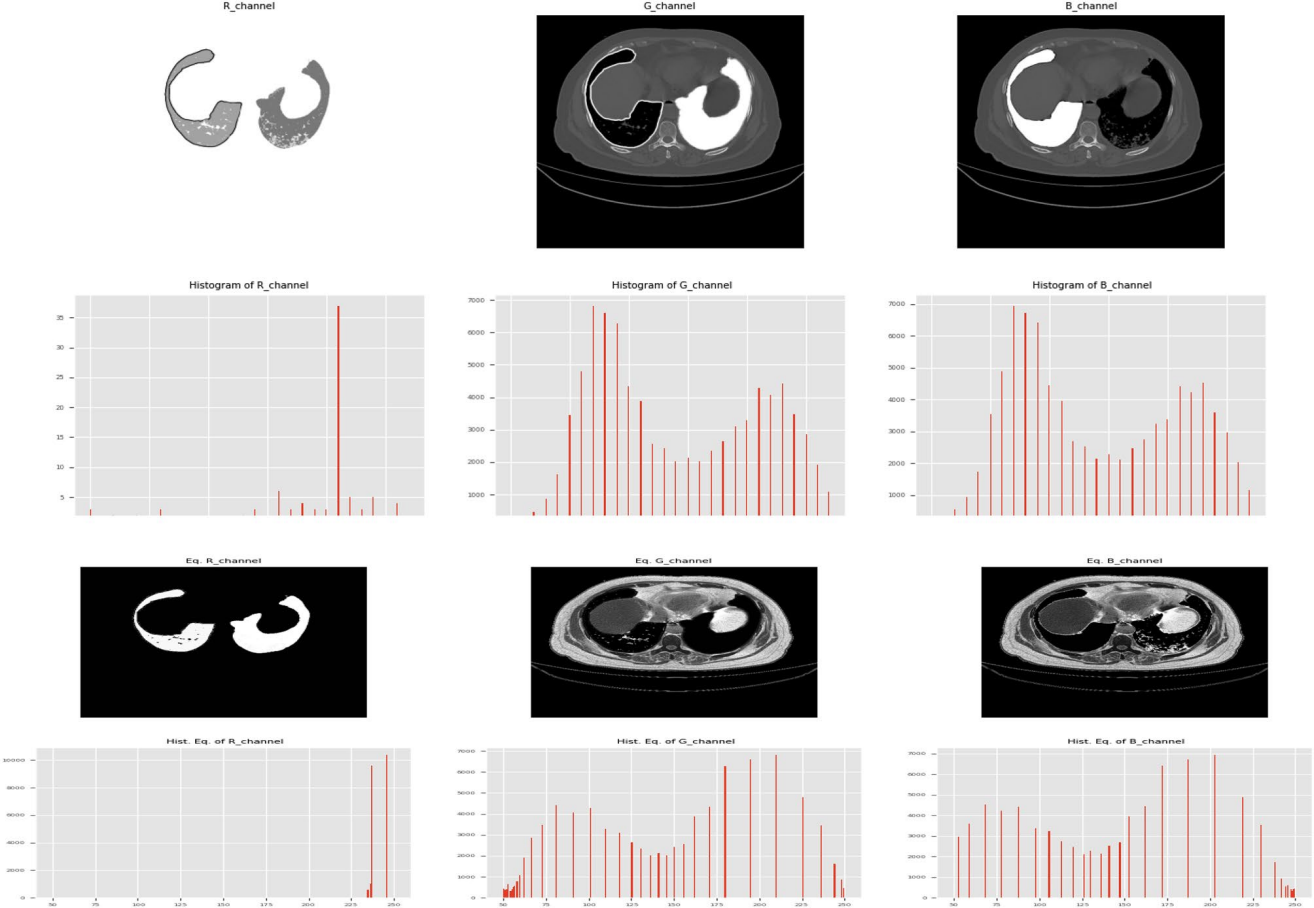
An example of the RGB image channel before and after applying the CLAHE.

Pre-processing is the initial procedure that takes place before the dataset is fed into the deep learning model. The CT scan image must be resized since the lungs are so big. To speed up training and testing, the data has been scaled to 128 × 128 instead of using large images as usual. When handling medical images, extreme caution must be taken because medical images are noisy and must be cleaned up before feeding them to the model; otherwise, the model would pick up on the noise^66,67^. An effective pre-processing phase is needed to improve the model's performance by removing noise and artifacts that might hinder the model's ability to learn and generalize. Medical images frequently suffer from two contrast issues, such as noise and intensity inhomogeneity; so, we used the Contrast Limited Adaptive Histogram Equalization (CLAHE) method, which was proposed in^68^, to improve the contrast of the obtained images. Figure 6 displays a sample of the lung CT scan image's CLAHE result for each RGB image channel before and after applying the CLAHE.

To enlarge the dataset for deep learning processes, a data augmentation strategy must be employed to acquire more images, given that Kaggle's CT scan images collection contains fewer than necessary images. As previously mentioned, the majority of approaches have attempted to address the data shortage using a variety of data augmentation techniques; however, there is no conclusive proof of the efficacy of data augmentation in real-life and live images for the detection of COVID-19. In contrast, we enlarged the dataset into 3138 × 3 without the need for any augmentation methods by employing various pre-processing image processing techniques to split the image into three RGB image channels, which are fed into three separate U-Net for each channel. The use of three RGB image channels provides further information to increase infection detection. The convolution blocks of the U-Net contain two convolution layers, and each layer has a different number of filters, which enhances U-Net segmentation, and reduces the number of parameters. The various image processing techniques used include thresholding, resizing, inversing image colors, using histogram equalization, and heat map as a data visualization tool. A density heat map was applied to the lung images to visualize the lung part in RGB color form. This provides more details that facilitate the detection of infection in the lungs. Our idea was motivated by the fact that mapping scalar values of medical images to colors is a helpful process that may be used to highlight features like bone density, tissue density, or blood flow, as discussed in^27,69^. The research created on COVID-19 always achieves high performance in lung segmentation and detection; however, COVID-19 infection segmentation and detection need more enhancement as they achieve low accuracy, as given in^48–52^. In the case of COVID-19, infected individuals often exhibit fever, which can be detected using thermal imaging and represented as a heat map^9,48^. Heat map is a graphical representation used to identify the regions of interest in the data by observing how colors change, and it is considered to be the best data visualization tool. It works with all image types, not just thermal images, such as those in^70,71^. To detect COVID-19 infection in the lungs, some researchers have used lung instruction. Others used grey lung images and depended on the power of the segmentation method, as in ^35^. The authors of^36^ highlighted the challenges faced in developing deep learning models for COVID-19 detection and diagnosis, such as the lack of large-scale datasets and the need for interpretability of the models. The following stages can serve as an overview of our proposed method's pre-processing phase:

**Figure 7:**
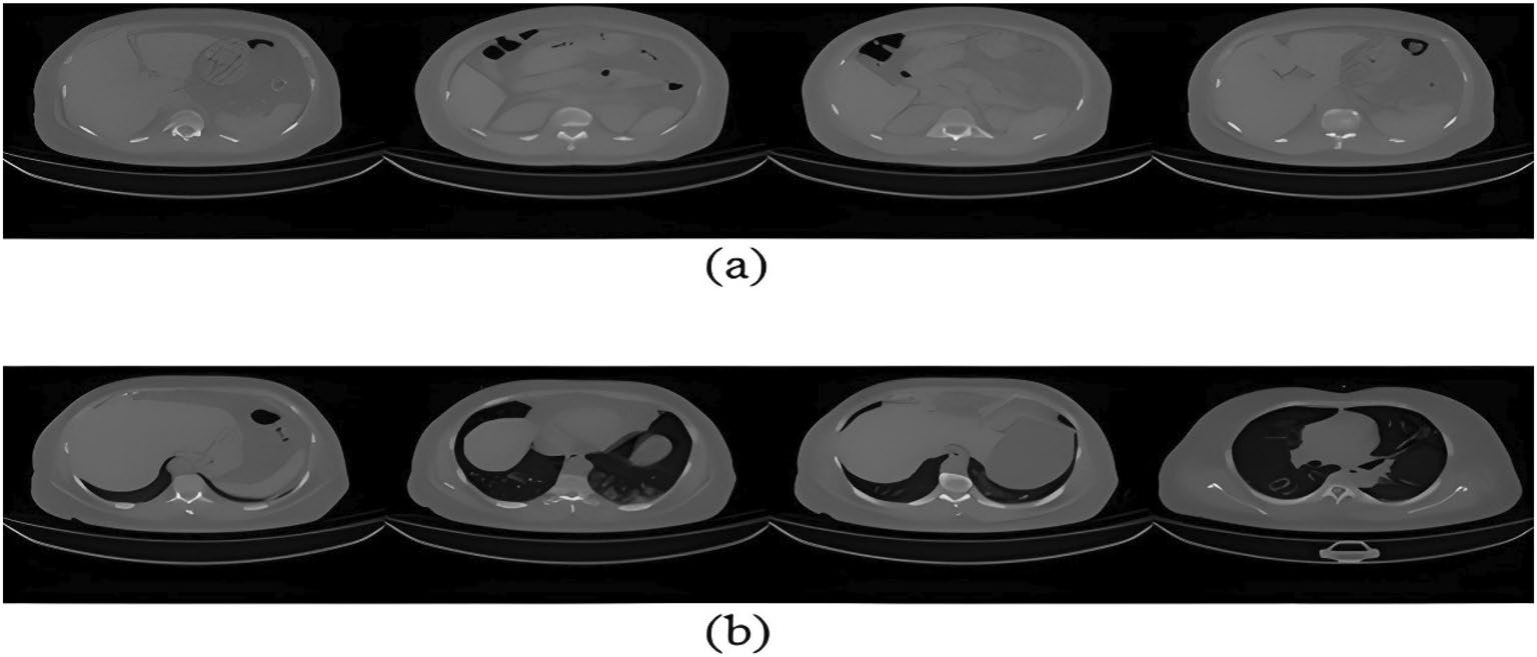
(**a**) Samples of images that were excluded; (**b**) samples of images that were included.

**Figure 8:**
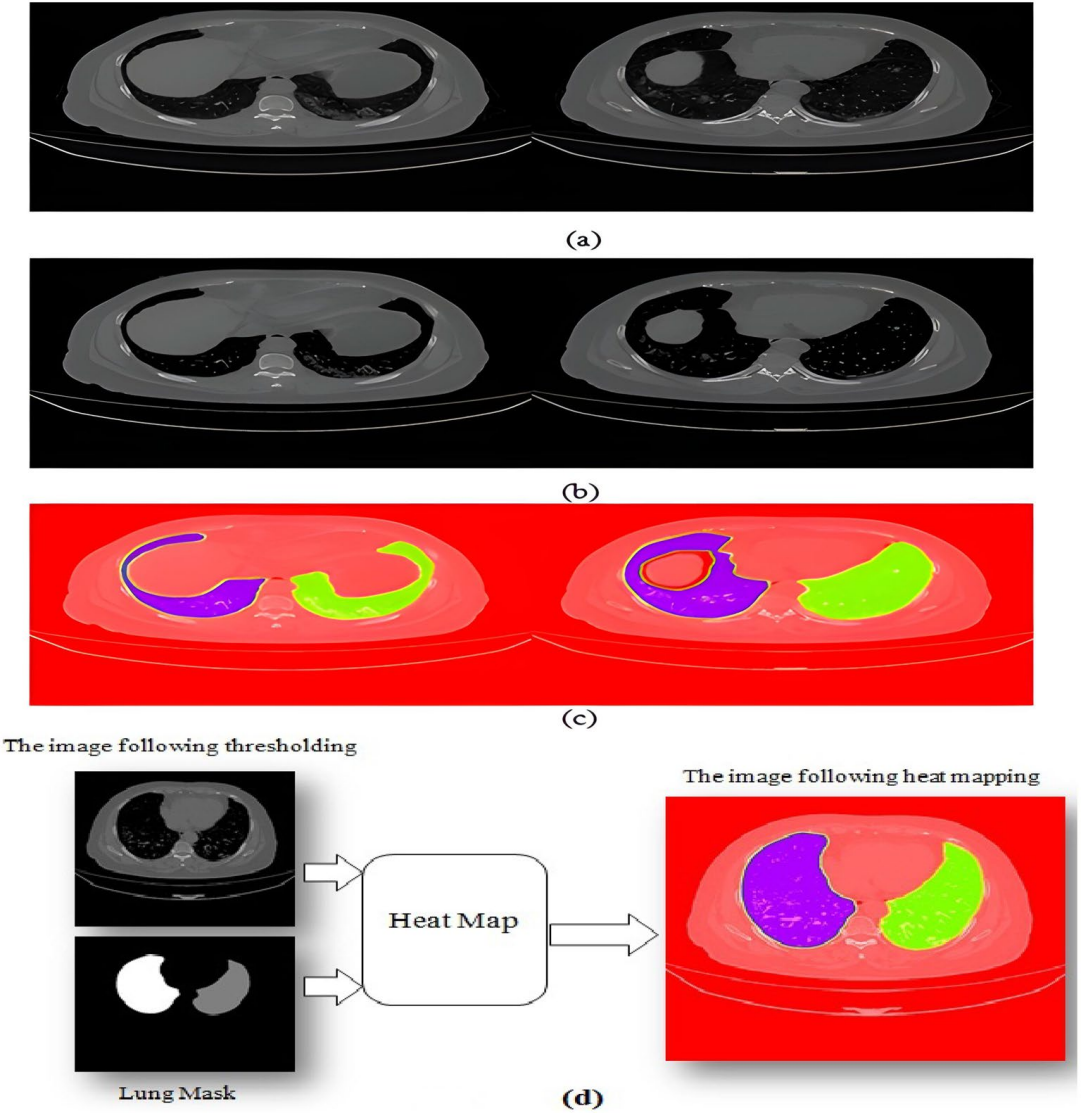
(**a**) A sample of CT scan images before thresholding, (**b**) a sample of those same images after thresholding, (**c**) a sample of images following the application of a heat-map, and (**d**) an illustration of heat map image (colored image) generation.

1. Eliminating artifacts and noise.
2. Remove all the images that don't depict lungs from the datasets (refers to Fig. 7).
3. Resize the dataset's images to match the dimensions of the input layer of the network $\left( {128 \times 128} \right)$
4. The images are given a threshold. This threshold is calculated by a simple equation given as follows: if pixel intensity is less or equal 50, make it zero; otherwise, don't change. This process simplifies the lung pictures by removing extraneous details. Figure 8a,b shows samples of images before and after thresholding.
5. After thresholding, create a density heat map^46^ by applying an RGB colour scheme to the area of interest. (i.e., lung). The original image is then overlaid with the heat map. Figure 8c shows the effect of applying the heat map on lung images. This step was quite helpful in getting rid of the infection in the lung area.

There is free software that may be used to construct heat maps, including the languages R for computing statistics and graphics, OpenLayers, Gnuplot, and Python, among others. In this study, we implemented the heat map using Python. Implementation and setup are discussed later in the "Result analysis and discussion" section. The thresholded images and masks of the lungs from the datasets, as shown in Fig. 8d, were used to create the heat map image (colored image), which is presented in Fig. 8c. The processes are given as follows:

1. The Gaussianblur, which uses the equation shown below, was initially applied to the mask image,
  $$G\left(x\right)=\frac{1}{2\pi {\sigma }^{2}}{e}^{-\frac{{x}^{2}}{2{\sigma }^{2}}}$$
  where x is the input image and $\sigma$ is the standard deviation of the Gaussian distribution of the image pixels. Using the OpenCV function, the implementation is as follows to create the blurred image:
  $${\text{blur }} = {\text{ cv2}}.{\text{GaussianBlur}}\left( {{\text{lung}}\_{\text{mask}},\left( {{5},{5}} \right),{\text{ cv2}}.{\text{BORDER}}\_{\text{DEFAULT}}} \right)$$
  where blur is the output image from Gaussianblur, and lung_mask is the mask of lung from the dataset.
2. We used the following OpenCV function to determine the color space that is applicable to the heat map:
  $${\text{heatmap}}\_{\text{img }} = {\text{ cv2}}.{\text{applyColorMap}}\left( {{\text{blur}},{\text{ cv2}}.{\text{COLOR}}\_{\text{BGR2RGB}}} \right)$$
  where heatmap_imag is the output of choosing the color space.
3. The following OpenCV function may be used to create the heat map's colored image:
  $${\text{Colored}}\_{\text{img }} = {\text{ cv2}}.{\text{addWeighted}}\left( {{\text{heatmap}}\_{\text{img}},{ 1},{\text{ threshold}}\_{\text{image}},{ 1},{ 1}} \right).$$

### Proposed algorithm

**Figure 9:**
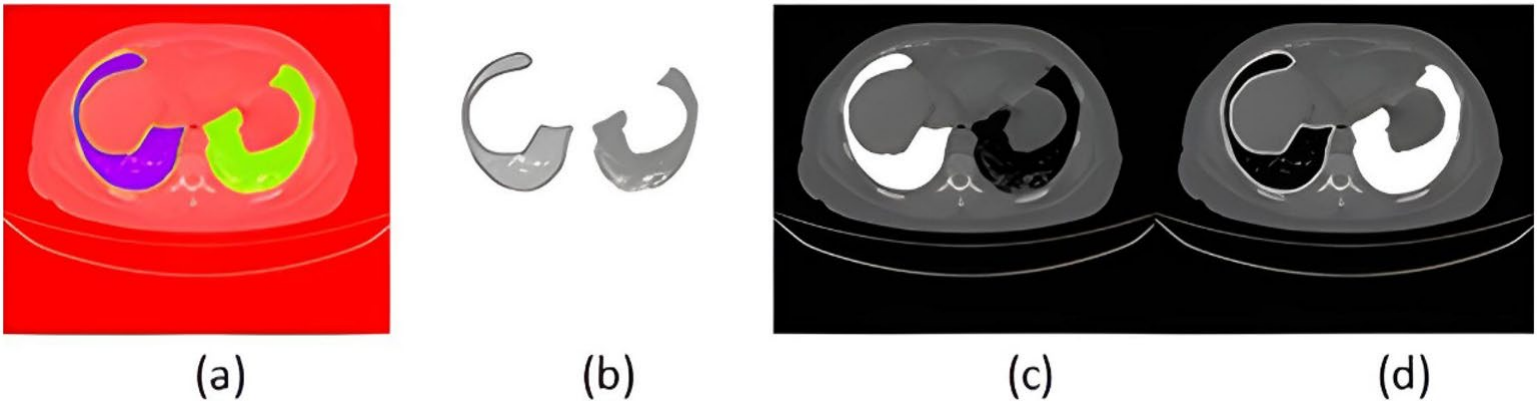
(**a**) Colored image, (**b**) red-channel, (**c**) blue-channel, and (**d**) green-channel.

**Figure 10:**
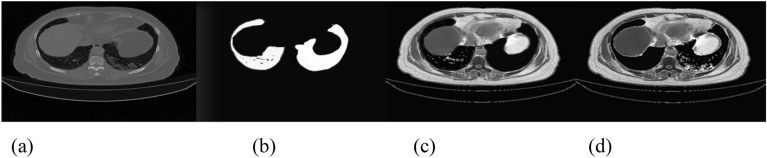
(**a**) Original image, (**b**) red-channel after histogram equalization and image inverse, (**c**) blue-channel after histogram equalization and image inverse, and (**d**) green-channel after histogram equalization and image inverse.

After the pre-processing phase, including the heat-map, the images are divided into three channels, namely, R-channel, G-channel, and B-channel. Then, a U-Net architecture based on CNN encoder and decoder approaches is applied to each of the RGB channels of the colored image for quick and accurate image segmentation to obtain the lung segmentation model. After visualizing the channel, we discovered that the red channel included the majority of the crucial data on infections and lung disorders. In the images, this information is rounded off with white areas. The white portion of the lung can have an impact on picture segmentation, while the green and blue channels can include information about infections. In order to perfectly clarify the in-image information, the image channels are processed using histogram equalization and the image inverse. Samples of the employed RGB channels are shown in Fig. 9. The examples of the images given in Fig. 10 were created using the image inverse and histogram equalization. Each channel passes into the U-Net architecture, and before classification, the segmented output is merged and fed into $1 \times 1$ convolution layer.

### Network architecture

**Figure 11:**
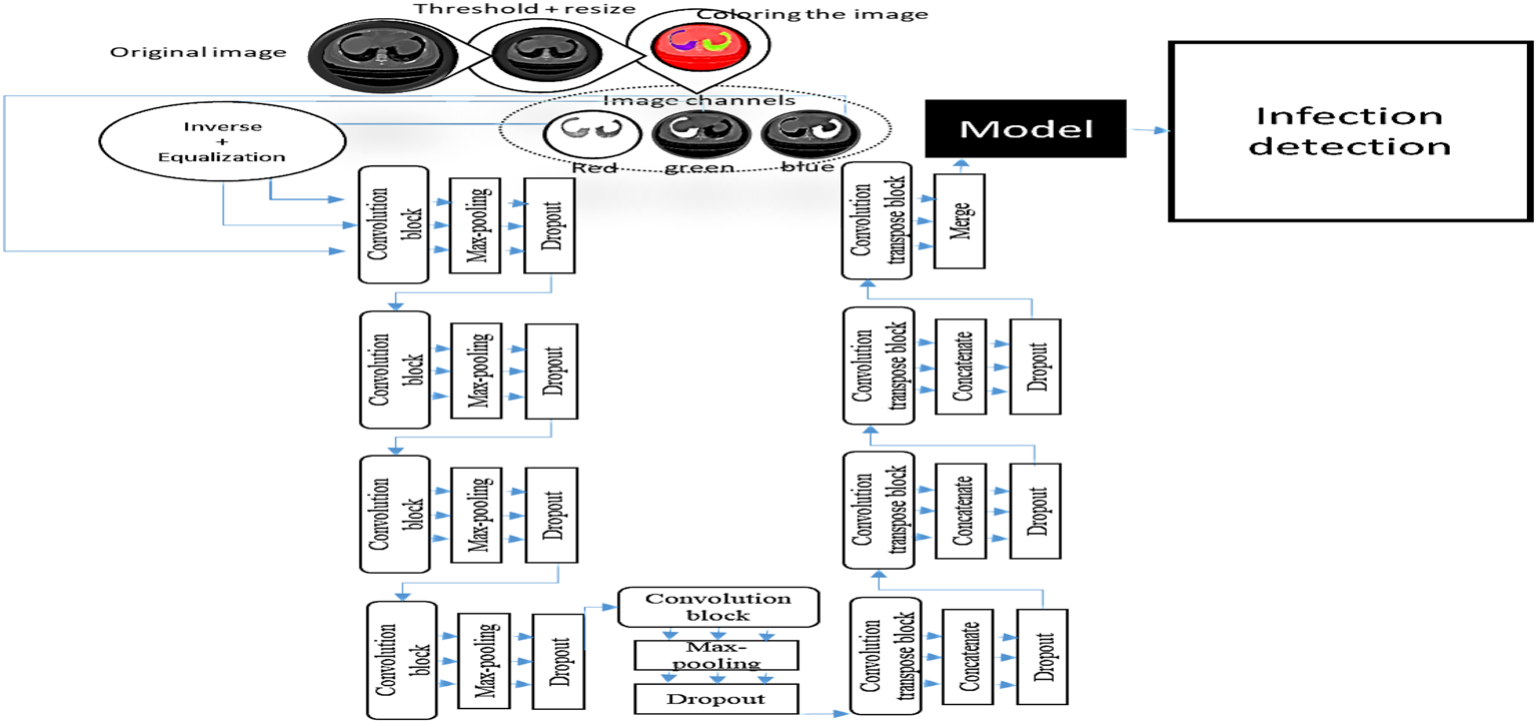
The detailed design of the network architecture.

The proposed approach contains three U-Nets. Each U-Net contains nine convolutional neural network blocks. Four blocks in the expanding path and five blocks in the contracting path. Two convolution layers with a filter size of $3 \times 3$ make up each convolution block. The number of filters in the first convolution layer in the convolution block was 50 and the number of filters in the second convolution layer was 25. To avoid overfitting in neural networks, we used dropout regularization after each convolution block in the U-net. During training, a dropout regularization strategy avoids overfitting by preventing any units from being codependent on one another. Dropout is often used to improve the performance (accuracy) of deep learning tasks on unknown datasets to avoid overfitting^72^. Our approach aims to avoid overfitting by minimizing both the batch size and feature count. This is accomplished by applying a max-pooling layer with a size of 2 × 2 and a dropout algorithm from the Python Keras package with a value of 0.01 to each convolution block in the U-net. An activation function called ReLU is then used. The dropout Python function that is used modifies the feature value that is larger than 0 by a factor of (1/1-0.01), while leaving the 0 feature value unchanged. The batch size was given a value of 32. In the classification layer, the sigmoid activation function is also applied, as shown below:

1 $$y = 1/(1 +{e}^{-x})$$

where y is the activation function's output, and stride in the used U-Net was equal to 2. Figure 11 shows the network architecture's complete structure.

## Result analysis and discussion

### Implementation and setups

Our system is implemented using the Python programming language with libraries such as TensorFlow, Keras, and open-CV. We ran the network using Keras and TensorFlow on a Dell laptop with an Intel(R) Core(TM) i5-1035G1CPU; generation 10, 8 GB of RAM, Windows 10, and a 64-bit operating system with an × 64-based processor. Experimental results were obtained for 20 CT scan datasets. The total number of images and masks used was 7040. After excluding closed lungs, 5,946 images were obtained. A total of 2973 were imaged with the lungs and 2973 with masks. There were 1783 images in the training set and 1190 images in the test set. Additionally, the masks had the same image numbers.

### Training and evaluation metrics

Image classification is comprised of two stages: training and testing. The dataset was divided into two groups: 70%of the images were used for training and 30% for the testing process. This division was randomly. The research community uses a variety of performance metrics to evaluate the efficiency of classification and segmentation algorithms. The F1-score, also known as the Dice coefficient, recall, accuracy, specificity, and precision, are some of these metrics. Four measurements are required to calculate these metrics and validate the proposed model: true positive (TP), true negative (TN), false positive (FP), and false negative (FN).

- *True positive (TP)*: represents the number of pixels being correctly identified in the segmentation tasks and the number of correctly predicted infected CTs in the classification task. Simply, it represents the correct classified images.
- *True negative (TN)*: denotes the number of non-lung/infection pixels being correctly identified as non-lung infection in the segmentation tasks and the number of correctly predicted healthy CTs in the classification task. Simply, it represents the images with a wrong class label and is classified to this wrong class.
- *False positive (FP)*: represents the number of non-lung/infection pixels being wrongly classified as lung/infection pixels in the segmentation tasks and the number of mistakenly predicted infected CTs in the classification task. Simply, it represents the wrong classified images.
- *False negative (FN)*: denotes the lung/infection pixels being wrongly classified as non-lung/infection pixels in the segmentation tasks and the number of mistakenly predicted healthy CTs in the classification task. Simply, it represents the images with a wrong class label and is not classified to this wrong class.

Based on the above four measurements, the performance metrics, which include accuracy, precision, sensitivity, specificity, and F1-score, were calculated as follows:

- *Accuracy*: The accuracy statistic counts the number of times a model predicts accurately over the whole dataset. It measures the ratio of correctly identified predictions divided by the entire prediction, and it is defined in the equation below:
  2 $$\mathrm{Accuracy }=\frac{{\text{TP}}+{\text{FN}}}{{\text{TP}}+{\text{FP}}+{\text{FN}}+{\text{TN}}}$$
- *Precision:* The ratio of correctly predicted positive observations to all positively expected observations is known as precision**.** Given in the equation below:
  3 $$\mathrm{Precision }=\frac{{\text{TP}}}{{\text{TP}}+{\text{FP}}}$$
- *Recall (also known as sensitivity):* It is the proportion of true positives correctly predicted by the model. It is the percentage of accurately predicted positive observations among all observations in the current class. Given in the equation below:
  4 $$\mathrm{Recall }=\frac{{\text{TP}}}{{\text{TP}}+{\text{FN}}}$$
- *Specificity*: it is the proportion of true negatives correctly predicted by the model**:**
  5 $$Specificity = \frac{TN}{{TN + FP}}$$
- *F1-score (Dice coefficient):* It is the proportion of the predictions to the actual data that overlaps. Its value ranges from 0 to 1 and the higher the value, the more accurate the segmentation. It is provided in the equation below:
  6 $${\text{F}}1-\mathrm{score }=\frac{{\text{TP}}}{{\text{TP}}+\frac{1}{2}({\text{FP}}+{\text{FN}})}$$

**Figure 12:**
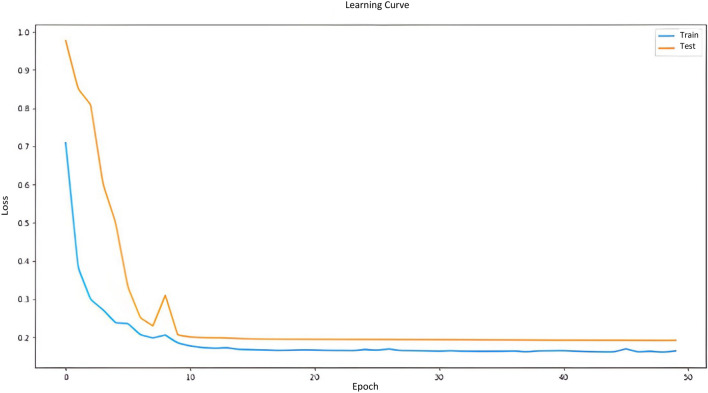
Learning curve.

**Figure 13:**
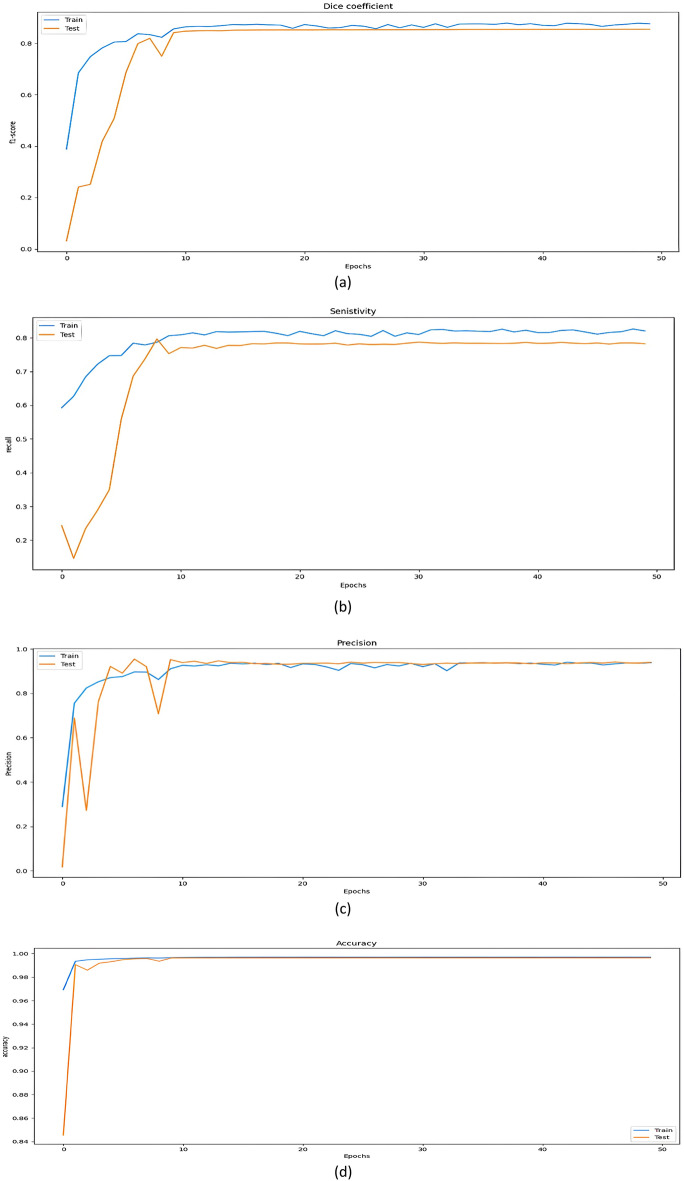
Performance results through the different evaluation metrics: (**a**) dice coefficient, (**b**) sensitivity, (**c**) precision, and (**d**) accuracy.

**Figure 14:**
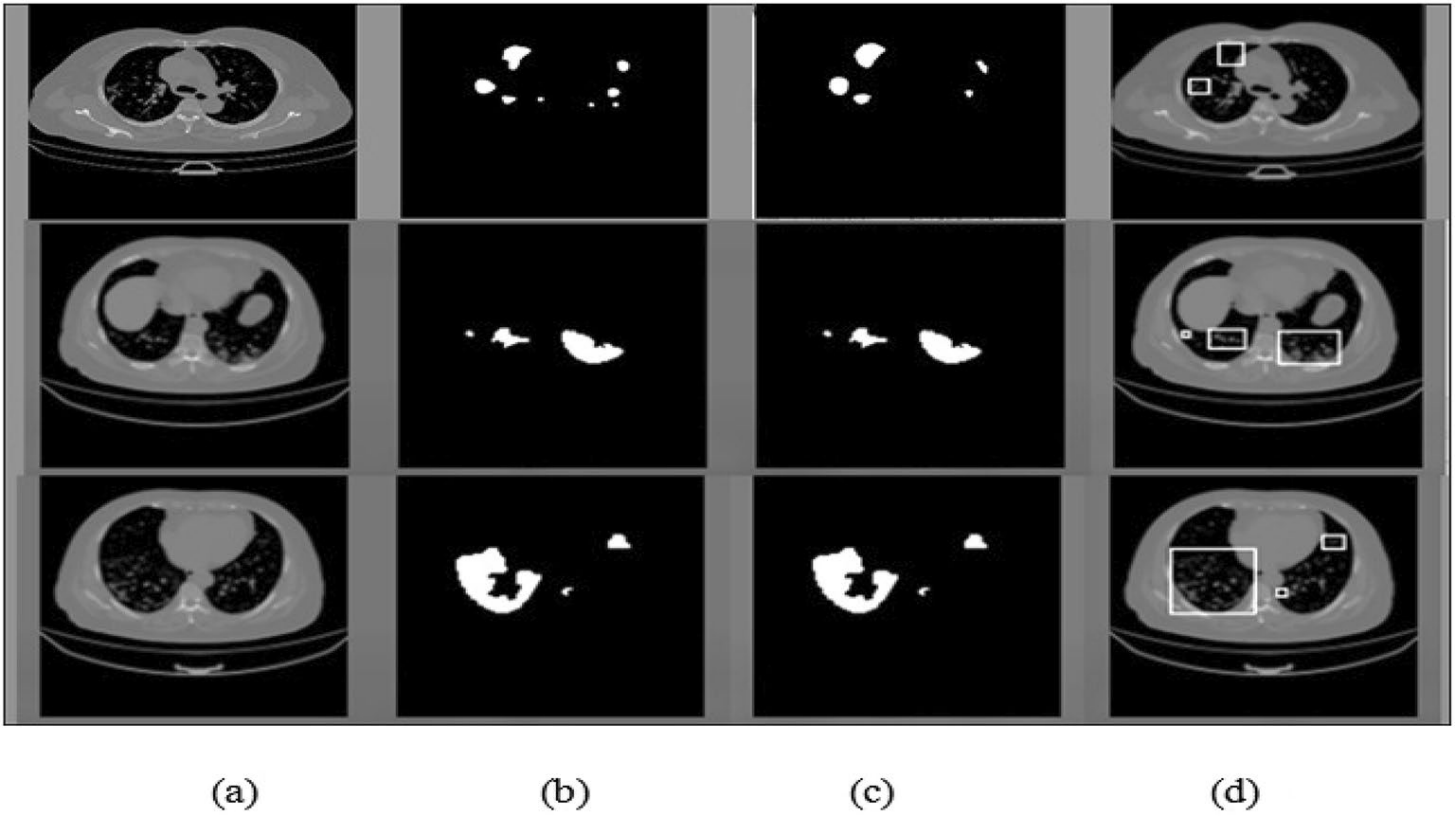
Experimental results (**a**) original image, (**b**) lung mask, (**c**) segmentation results, and (**d**) detection.

The evaluation metrics findings for our proposed approach are shown in Table 1, and they demonstrate good performance. According to the learning curve illustrated in Fig. 12 which is based on the training and testing losses across 50 epochs, such good performance is confirmed. From this curve, we found that the accuracy improved during the 50th epoch and that the loss from epoch 9 did not significantly change. Figure 13 shows the evaluation metrics results of the proposed approach in terms of accuracy, precision, sensitivity and dice coefficient. Experimental results in the segmentation and detection process are demonstrated in Fig. 14.

**Table 1:** Evaluation criteria for our proposed algorithm.

Sensitivity	Precision	Dice coefficient	Accuracy (%)
0.83	0.87	0.85	99.71

### Ablation study and comparative analysis

In deep learning, we frequently use models that are composed of a variety of components, each of which has a significant impact on the overall performance, so, it is crucial to provide a method for evaluating the contribution of these components to the overall model. This is where the idea of an ablation study comes in, when specific network components are eliminated in order to better understand the behavior of the network. In other words, an ablation study, which is used to quantify causality, is a simple method to look into the causes of those components. We conducted an ablation study to demonstrate the model's robustness by eliminating a part of the proposed approach's components, referred to as “mode”, while leaving the others unchanged. The significance of each component (Red channel, Green Channel, Blue channel, histogram equalization, among others), used in the proposed approach is presented in Tables 2 and 3, with the help of the evaluation metrics (e.g., sensitivity, precision, dice coefficient, and accuracy) to highlight their impact on the segmentation performance for COVID-19 detection.

**Table 2:** Effects of the proposed components on the model's performance (without heat map).

Mode	Infection segmentation			
	Sensitivity	Precision	Dice coefficient	Accuracy (%)
Removal of the red channel and its U-net from the system	0.71	0.72	0.72	99.56
Removal of the green channel and its U-net from the system	0.68	0.70	0.69	99.52
Removal of the blue channel and its U-net from the system	0.71	0.74	0.72	99.57
Our proposed approach without removing	0.66	0.76	0.71	99.57

**Table 3:** Effects of the proposed components on the model's performance (with heat map).

Mode	Infection segmentation			
	Sensitivity	Precision	Dice coefficient	Accuracy (%)
Removal of the red channel and its U-net from the system	0.7	0.8	0.74	99.52
Removal of the green channel and its U-net from the system	0.63	0.64	0.63	99.27
Removal of the blue channel and its U-net from the system	0.72	0.76	0.74	99.49
Our proposed approach without removing	0.83	0.87	0.85	99.71

Table 4 shows a comparative study of our proposed framework with other existing ones that use the same dataset and are based on training time, error rates and dice coefficient metrics. We found that our method, which operates on 128 × 128 images, outperforms existing methods that use larger 512 × 512 image sizes. Although we use the same U-Net and dataset as other frameworks, our proposed approach has a distinct structure and better accuracy. For instance, the authors of^63^ have achieved a dice coefficient rate of 76% and used data augmentation by increasing the data by 15% through random intensity to enhance the results of the 3D U-Net. On the contrary, our proposed approach achieved a dice coefficient rate of 0.85 without the need for any augmentation strategies. Scholars of^65^ used two cascaded residual attention inception U-Net (RAIU-Net) models and achieved a dice coefficient rate of 0.81, while study^64^ used edge-enhancing diffusion filtering (EED) to improve the contrast and intensity homogeneity of the infection areas with a dice coefficient rate of 0.78. Finally, the authors of^62^ suggested a U-Net for COVID-19 detection and trained it using 80% of the data, with an attainable dice coefficient rate of 0.67. It is important to point out that the research studies^63–65^ separated their data into 70% for training and 30% for testing, much like we did in our proposed methodology.

**Table 4:** A comparative study of our proposed framework and other existing ones.

Dataset	Sources/references	Dice coefficient	Error rate	Training time
Kaggle Dataset^32^	Our proposed approach	0.85	0.29%	18.7 s/image
	^65^	0.81	3.6%	NA
	^64^	0.78	Not applicable (NA)	NA
	^63^	0.76	NA	NA
	^62^	0.67	NA	NA

### Limitations

There are some potential limitations that need to be highlighted, one of which is the limited availability of datasets for training models due to the novelty of COVID-19 disease. The proposed technique was implemented using Kaggle, the first publicly available COVID-19 CT scan images dataset repository, available at https://www.kaggle.com/datasets/andrewmvd/covid19-ct-scans. However, additional publicly accessible datasets are now available and summarized in^23^. Also, we would need further training with larger and more adaptable datasets to compare the outcomes in order to be more effective in clinical practice. On a larger dataset, the algorithm's accuracy could differ. Another limitation in our research is that we did not focus on the different levels of infection severity in the lung CT scan images of COVID-19. Moreover, the accuracy of COVID-19 identification may also be impacted by a lack of diverse data, false positive and negative measurements, applying the method to different hyper-parameter configurations (e.g., epoch size, batch size, activation function, optimizer techniques, etc.), among others. All of these aspects could be useful for achieving a more reliable model for practical applications.

## Conclusion

The COVID-19 virus, a threat that exists on a worldwide scale, has an influence on millions of people's lives. The fight against this pandemic depends on the earliest detection of COVID-19 symptoms. To aid in early illness detection and disease prevention, deep learning algorithms have been trained to identify and classify lung images. In this paper, we propose a new approach that combines image processing, data visualization, and deep learning (DL) techniques, particularly U-Net architecture, to accurately detect COVID-19 infections with the existing COVID-19 CT scans publicly available at Kaggle dataset repository. The majority of earlier research that used the same dataset for training and testing has attempted to address the data shortages using various data augmentation techniques, since Kaggle's collection of CT scan images has fewer images than necessary. However, data augmentation in real-life and live images hasn't been demonstrated to be particularly helpful in detecting COVID-19. On the other hand, our approach uses a variety of pre-processing image-processing techniques to divide the image into red, green, and blue (RGB) channels, which are then fed into three separate U-Nets for each RGB image channel. This method directly expands the original dataset without the need for any augmentation strategies. The three RGB channels were used to increase the feature information, consequently improving infection detection by employing a lot of data instead of the grey image, which only comprises one channel; this division helps with infection detection. The various image processing techniques used include thresholding, resizing, inversing image colors, using histogram equalization, and heat map. A heat map is an effective method for data visualization that can be used to envision the lung image in RGB color space and identify the region of interest in data by noticing how colors change. We evaluated the performance of our approach using accuracy, precision, sensitivity, specificity, and dice coefficient metrics, and we found that our method showed good performance on 128 × 128 images. However, other algorithms were able to improve their segmentation by using larger 512 × 512 images.

## Data Availability

All data generated or analyzed during this study are included within the article.

## Code availability

The overall source code used in this study is available from the corresponding author upon request.
