# Author Correction: COVID-19 infection segmentation using hybrid deep learning and image processing techniques

**DOI:** 10.1038/s41598-024-53425-1

**Published:** 2024-02-05

**Authors:** Samar Antar, Hussein Karam Hussein Abd El-Sattar, Mohammad H. Abdel-Rahman, Fayed F. M. Ghaleb

**Affiliations:** Computer Science Division, Department of Mathematics, Faculty of Science, Ain Shams University, Abbassia, Cairo, 11566 Egypt

Correction to: *Scientific Reports* 10.1038/s41598-023-49337-1, published online 20 December 2023

The original version of this Article contained an error in the name of the author Mohammad H. Abdel-Rahman which was incorrectly given as Mohamed H. Abd-Rahman.

The original Article has been corrected.

